# Forty-Fifth Annual Report of the General Board of Commissioners in Lunacy for Scotland

**Published:** 1903-07-18

**Authors:** 


### FORTY-FIFTH ANNUAL REPORT OF THE GENERAL BOARD OF COMMISSIONERS IN LUNACY FOR SCOTLAND.

ON January 1st Scotland had 16,658 insane persons registered as such, and this number does not include lunatics maintained in their own homes by their natural guardians. It is shown that the above number was distributed as follows:—4,286 in royal asylums, 7,373 in district asylums, 125 in private asylums, 502 in parochial asylums, 1,153 in workhouses, 2,771 in private dwellings, 51 in prisons, and 397 in training schools. If these numbers be contrasted with the numbers for the year 1858 it will be seen that the lunatics in Scotland have increased since that year by 186 per cent., and showing a net increase of 10,834 insane persons, and further that last year shows an increase of 370 over the previous year. If the average for the past 10 years be taken, the year 1902 exceeded that average by 74. When it is learnt that the increase in the population of Scotland ince 1858 has only been 50 per cent., it is clear that not smuch comfort can be gleaned from these figures.

There are two notable statements in this Scottish report which deserve careful reflection on the part of the English ratepayer. The first is that all the pauper patients who are in institutions at all are confined in public asylums, and that 2,642 of the patients are in private dwellings, whereas in England with a total of 110,713 insane persons there are 1,172 pauper lunatics in private asylums where they are maintained at a much higher cost than if in asylums for their counties, and all this loss to the nation has accrued because the authorities did not provide accommodation in time. All experience in these matters has demonstrated that where county councils have not anticipated the needs of their districts they have lagged behind them. Again the number of outdoor pauper lunatics in England is only 5,569. Verily they manage these things better in Scotland.

At present there is some talk that a modification may take place in England as regards private patients, or rather as regards those incipient cases of insanity which peremptorily require medical treatment; but not necessarily asylum treatment. It is cruel to brand these cases as registered lunatics until some trial has been made at effecting a cure; and we may, for the information of our readers, copy the conditions under which these and other cases are treated in Scotland. An insane patient may there be kept in a private house if he is certified by a registered medical practitioner that he is afflicted with a malady that is not confirmed and that it is expedient to treat him in a private dwelling for six months. This is a very sensible and proper provision. But if insane for more than a year or is subjected to compulsory confinement in the house; or if he possess property placed under curatory by a court of law, he would, in either of these cases, come under the official cognisance of the commissioners.

In any public asylum the number of patients in residence will depend upon the admission rate, the discharge rate, and the death rate. The first and the last cannot be controlled, but the manner in which the discharges are carried out will modify that terrible unending accumulation of asylum lunatics, because when every case has been discharged recovered which can fairly be brought under that category there remains a large proportion who, in the words of the Scottish commissioners, neither need asylum care nor are benefited by it. And it is by looking up these cases and inducing their friends to undertake charge of them that something might be done to relieve congested asylums, and perhaps to increase the happiness of those patients so discharged. But in England, at least, the ever-recurring need of additional asylum accommodation is in part owing to the action of the four-shilling capitation grant which induces the transfer of workhouse imbeciles to county asylums. Here, however, the surroundings of the imbecile are distinctly improved; but the rates are drawn on to obtain that improvement.

To those interested in asylum statistics, this Blue Book is well worth a careful study. It is not easy to put much freshness into a statistical work which has to make its appearance annually, but fresh points will generally be found in these Scottish reports.

